# The Stourfield Park Sanatorium, Pokesdown, Bournemouth

**Published:** 1901-10-19

**Authors:** 


### The Institutional Workshop.

### THE STOURFIELD PARK SANATORIUM, POKESDOWN, BOURNEMOUTH.

Bournemouth may be reached in a little over two hours from Waterloo, and for all practical purposes Bournemouth, Boscombe, and Pokesdown may be regarded as one and the same town. The three or four miles between them may be compassed by train, or cab, or omnibus, or even by motor-car, and any one of these will land the visitor within a minute or two of the sanatorium. This is a handsome building, new a few years ago, and is surrounded by about ten acres of private grounds. Immediately adjoining are pine woods and walks innumerable, and within ten minutes is the sea. For a long time the air of Bournemouth has been deemed curative, or alleviative, in lung diseases, and it was to be expected that open-air sanatoria would spring up in a locality so well suited for the treatment of consumption, so favourably known, and so easily accessible from London. As already said, Stourfield Park Sanatorium is almost new. This fact, joined with its admirable site, the facility with which it can be reached, the brightness of its rooms, and the variety of its surroundings, render it peculiarly suitable for successfully carrying out the modern treatment. On the occasion of our visit the large majority of the patients were either cured or convalescent.

The sanatorium is under charge of the resident physician, Dr. Thomson, and as there are only 37 beds for invalids, he is able to give each case the most careful individual attention. A series of good rules has been framed by Dr. Thomson for the guidance of the patients.

The Sanatorium contains six sitting-rooms and a large conservatory. The dining-room and the drawing room are very well lighted and brightly decorated, and these remarks are applicable to by far the larger number of bedrooms. On the kitchen department much of the regularity and success of any institution will depend. Here it is large, extremely clean, and has every essential requisite for efficient management. Speaking generally, the first and second floors are given up to the patients, and the third floor to the staff. In some cases the bedrooms have hexagonal bays of size sufficient to place a bedstead in, and so provide the sleeper with an abundance of fresh air In other rooms the windows run down to the floor and open on to balconies. This arrangement of window is an admirable one, and ought to be adopted in all sanatoria for consumption as it ensures that the floor of the room shall be swept by a current of air, and if to this be added an open door or an open fanlight over the door, there can be no likelihood of stagnation of air in any part of the room. In nearly all the bedrooms at Stourfield Park the upper segments of the windows are made to open on the casement principle.

On the lawn are fourteen kiosks of admirable construction. Apparently these are used by day only, and it seems a pity that some of them should not be converted into sleeping-rooms for which they are so well adapted.

The arrangements for bathing are unusually good, and include plunge, shower, and douche baths. A wide corridor traverses the entire length of the building on each floor and can be made use of as an efficient ventilator. The warming is by open fireplaces, but hot-water coils are placed in most of the rooms for use when necessary. The electric light is used throughout.

Of course the diet is liberal. Sterilised milk is given at 6 a.m., breakfast at 8.30, dinner at 1.30, tea at 4, supper at 6.30, and milk at 9. As pointed out by Dr. Thomson, the food is specially ordered to assist the cure of the patients in the shortest period, and the patient should exert every power of will to eat the quantity prescribed. Certain it is that the gain in weight under this diet is wonderful. At least one hour's rest is insisted on before each meal.

The question of visiting patients in sanatoria is one not always easy to solve. The friends of the sick are too often unreasonable in speech and action, and frequently the patient's cure is delayed by the visit. We cordially agree with Dr. Thomson's rule on this subject, and we hope the friends of patients will be content to visit them very occasionally, but we doubt if they will. As the "cure" is usually not more than a few months in duration, there is no reason why the advice should not be taken.

The fees are four guineas a week, and this sum includes medical attendance and ordinary nursing.

